# Parent-adolescent communication, self-esteem, and suicidal ideation in Chinese adolescents: evidence from a four-wave longitudinal study

**DOI:** 10.3389/fpsyt.2026.1909330

**Published:** 2026-08-06

**Authors:** Kun Hu, Bin Li

**Affiliations:** 1 Department of Psychological Counseling, Changsha Social Work College, Changsha, China; 2 Party and Government Office, Changsha Social Work College, Changsha, China

**Keywords:** cross-lagged panel model, parent-adolescent communication, random-intercept cross-lagged panel model, self-esteem, suicidal ideation

## Abstract

**Background:**

Previous studies have linked parent–adolescent communication and self-esteem to adolescent suicidal ideation; however, less is known about how these relationships unfold over time, whether maternal and paternal communication play different roles, and whether these associations reflect stable between-person differences or within-person changes.

**Method:**

Cross-lagged panel models (CLPM) and random-intercept cross-lagged panel models (RI-CLPM) were used to examine the longitudinal relationships among parent-adolescent communication, self-esteem, and suicidal ideation. A four-wave longitudinal survey with six-month intervals was conducted among 1,036 Chinese adolescents (47.4% female; *M*_age_ = 13.39 ± 0.54 years). Parent-adolescent communication, self-esteem, and suicidal ideation were assessed using validated self-report measures.

**Results:**

The CLPM results indicated bidirectional associations between self-esteem and suicidal ideation and showed that both father–adolescent and mother–adolescent communication were associated with subsequent self-esteem and suicidal ideation across time. Self-esteem also accounted for part of the longitudinal associations between parent–adolescent communication and suicidal ideation. The RI-CLPM results further showed that, at the within-person level, only mother–adolescent communication predicted subsequent decreases in suicidal ideation, whereas father–adolescent communication showed no significant effects. Self-esteem consistently predicted subsequent suicidal ideation across time, whereas evidence supporting reverse effects was relatively limited.

**Conclusion:**

This study advances understanding of the dynamic relationships among parent–adolescent communication, self-esteem, and suicidal ideation. The findings suggest that maternal and paternal communication may play different roles in adolescent mental health and highlight positive parent–adolescent communication and self-esteem as potential targets for adolescent suicide prevention.

## 1 Introduction

Adolescence is a critical developmental period characterized by substantial physical and psychological changes and is also a peak period for the emergence of psychological problems. According to the World Health Organization, suicide is the second leading cause of death among individuals aged 15-29 (1). Suicidal ideation, referring to thoughts about ending one’s own life, is widely recognized as an important precursor to suicidal behavior (2). In China, approximately 18% of adolescents report suicidal ideation prior to suicide attempts (3), and adolescents with suicidal ideation are substantially more likely to attempt suicide later in life (4). Therefore, understanding the mechanisms underlying adolescent suicidal ideation is important for suicide prevention.

According to Family Systems Theory, the family functions as an interconnected system in which interactions among family members influence individual development (5). From this perspective, parent-adolescent communication represents an important pathway through which family processes may shape adolescents’ psychological adjustment. Parent-adolescent communication, defined as the process through which parents and adolescents exchange information, share emotions, and express needs (6, 7), has consistently been linked to adolescent suicidal ideation (8, 9). Adolescents who experience difficulties communicating with their parents are more likely to feel lonely, hopeless, and unsupported, thereby increasing the risk of suicidal ideation (10). Conversely, supportive parent-adolescent communication may buffer the negative effects of stressful experiences and reduce suicide risk (11, 12).

However, previous studies have often combined father-adolescent communication and mother-adolescent communication into a single construct of parental communication (13, 14). Fathers and mothers may play different roles within the family system and interact with adolescents in different ways (15). Mothers are generally more involved in emotional expression and daily caregiving, whereas fathers tend to provide greater autonomy support and problem-solving guidance (16, 17). These differences suggest that father-adolescent communication and mother-adolescent communication may have distinct associations with adolescent suicidal ideation. Therefore, distinguishing these two forms of communication may provide a more nuanced understanding of family influences on adolescent suicide risk. Based on this, the present study hypothesized that both father-adolescent communication and mother-adolescent communication would negatively predict adolescent suicidal ideation.

In addition to family factors, individual characteristics also play an important role in adolescent suicidal ideation. Self-esteem, defined as an individual’s overall evaluation of self-worth, has consistently been associated with suicidal ideation (18, 19). The vulnerability model suggests that low self-esteem increases susceptibility to psychological difficulties, whereas the scar model proposes that psychological distress may subsequently undermine self-esteem (20, 21). Extending these perspectives to suicidal ideation, adolescents with lower self-esteem may be more vulnerable to suicidal thoughts because they perceive themselves as less capable of coping with challenges and more hopeless about the future (22). At the same time, persistent suicidal ideation may further damage self-esteem by reinforcing negative self-perceptions (23, 24). Together, these perspectives suggest that self-esteem may function as both a vulnerability factor and a psychological consequence in the development of suicidal ideation, highlighting the importance of examining their longitudinal and reciprocal associations.

Notably, self-esteem may also serve as an important mechanism linking family relationships to suicidal ideation. According to symbolic interactionism, individuals develop their self-concepts through social interactions and the feedback then received from significant others (25). Within the family context, parents represent important sources of social evaluation, and adolescents may gradually internalize parental acceptance, support, or rejection into their own self-perceptions. Therefore, supportive parent-adolescent communication may foster positive self-evaluations and enhance adolescents’ sense of self-worth, whereas poor-quality communication may undermine self-esteem (26). Longitudinal evidence has further shown that positive parent-adolescent communication predicts subsequent increases in adolescent self-esteem (27). Therefore, self-esteem may mediate the relationship between parent-adolescent communication and suicidal ideation.

Although previous studies have provided evidence for associations among parent-adolescent communication, self-esteem, and suicidal ideation, several limitations remain. First, most studies have not distinguished father-adolescent communication from mother-adolescent communication, potentially obscuring their unique contributions. Second, evidence regarding the mediating role of self-esteem has largely relied on cross-sectional designs, limiting conclusions regarding temporal relationships. Third, previous longitudinal studies have predominantly used traditional cross-lagged panel models, which do not distinguish stable between-person differences from within-person fluctuations over time.

Guided by Family Systems Theory, Symbolic Interactionism, and the vulnerability/scar models of self-esteem, the present study aimed to develop a more comprehensive understanding of how parent-adolescent communication is associated with adolescent suicidal ideation. Using four-wave longitudinal data to construct both traditional cross-lagged panel models (CLPMs) and random intercept cross-lagged panel models (RI-CLPMs), allowing for a more comprehensive examination of the longitudinal associations among parent-adolescent communication, self-esteem, and suicidal ideation. Specifically, we examined (1) whether father- and mother-adolescent communication showed differential associations with suicidal ideation, (2) whether self-esteem mediated these longitudinal associations, and (3) whether these associations remained significant after separating between-person differences from within-person fluctuations.

## 2 Methods

### 2.1 Participants and procedure

Participants were eighth-grade students recruited from two public middle schools in Hunan Province, China. A convenience sampling approach was adopted. First, two middle schools were selected based on their willingness to participate and their accessibility to the research team. Subsequently, all eighth-grade students from the participating schools were invited to take part in the study.

The inclusion criteria were: (1) being enrolled as an eighth-grade student at one of the participating schools; (2) being able to understand and complete the questionnaire independently; and (3) providing informed assent with parental or legal guardian consent. Before data collection, ethical approval was obtained from the Ethics Committee of Changsha Social Work College. Written informed consent was obtained from parents or legal guardians, and adolescents provided their own assent before participation. Given that suicidal ideation was assessed in this study, additional procedures were implemented to protect participants’ safety while protecting their privacy and confidentiality. During the consent process, participants and their parents or legal guardians were informed that their responses would be kept confidential, except in situations involving serious concerns about participant safety. After completing the questionnaires, participants received a brief standardized debriefing, including reassurance that psychological difficulties are common and information about available mental health resources. This debriefing was not intended as a psychological intervention and was provided uniformly to all participants to minimize any potential influence on subsequent assessments. If a participant was identified as being at potential risk of suicide based on their responses, appropriate safety procedures were followed while minimizing disclosure of personal information. When necessary, relevant school professionals were contacted to facilitate further support in accordance with ethical requirements.

A total of four longitudinal surveys were conducted using identical measurement instruments, with a six-month interval between each wave. At the first survey (T1), there were 1,131 participants. Subsequently, 117 participants were lost at the second survey (T2), 54 at the third survey (T3), and 84 at the fourth survey (T4). Participants who completed at least two surveys were included in the final analytic sample, which consisted of 1,036 adolescents aged 12 to 15 years. Among them, 491 (47.4%) were female and 545 (52.6%) were male; 829 (80.0%) resided in urban areas and 207 (20.0%) in rural areas. The mean age of the participants was 13.39 years (*SD* = 0.54). Attrition analyses using independent samples t-tests and chi-square tests revealed no significant differences between retained and dropped-out participants in terms of sex (χ² = 2.63, *p* = 0.11), age (t = −0.40, *p* = 0.69), father-adolescent communication (t = −1.81, *p* = 0.07), mother-adolescent communication (t = −1.46, *p* = 0.14), self-esteem (t = −1.51, *p* = 0.13), or suicidal ideation (t = 1.64, *p* = 0.10), indicating that there was no evidence of attrition bias. Missing data were handled using full information maximum likelihood estimation.

### 2.2 Measures

All measures used in the present study were previously validated Mandarin Chinese versions with established psychometric properties. The culturally adapted instruments have been widely applied in research involving Chinese adolescents, and their item wording is clear and appropriate for this population. Therefore, no pilot study was conducted prior to the main investigation.

Demographic information was collected using a self-report questionnaire developed by the research team. Participants were asked to provide basic demographic characteristics, including age, sex, and residential location.

#### 2.2.1 Parent-adolescent communication

Parent-adolescent communication was assessed using the scale originally developed by Barnes and Olson (7) and subsequently revised for the Chinese context by Wang et al. (28). The scale consists of two separate sub-scales: father-adolescent communication and mother-adolescent communication. Each subscale comprises two dimensions, open communication and problematic communication, with a total of 40 items. All items were rated on a 5-point Likert scale ranging from 1 (strongly disagree) to 5 (strongly agree). Items on the problematic communication dimension were reverse-scored such that higher total scores indicate higher quality of father-adolescent and mother-adolescent communication. In this study, the Cronbach’s α coefficients for father-adolescent communication across the four waves were 0.88, 0.87, 0.88, and 0.82, respectively; for mother-adolescent communication, the Cronbach’s α coefficients were 0.87, 0.87, 0.88, and 0.82, respectively.

#### 2.2.2 Self-esteem

Self-esteem was measured using the Rosenberg Self-Esteem Scale originally developed by Rosenberg (29) and subsequently revised by the Chinese context by Wang et al. (30), which consists of 10 items. All items were rated on a 5-point Likert scale ranging from 1 (completed disagree) to 5 (completely agree), which higher scores indicating higher levels of self-esteem. In the present study, Cronbach’s α coefficients for this scale across the four waves were 0.89, 0.90, 0.91, and 0.91, respectively.

#### 2.2.3 Suicidal ideation

Suicidal ideation was assessed using the originally developed by Osman et al. (31) and subsequently revised for the Chinese context by Wang et al. (32). The scale consists of 14 items, which were rated on a 5-point Likert scale ranging from 1 (never) to 5 (always). Higher total scores indicate higher levels of suicidal ideation. In the present study, the Cronbach’s α coefficients for this scale across the four waves were 0.92, 0.92, 0.93, and 0.88, respectively.

### 2.3 Statistic analysis

Data analyses mainly involved SPSS 26.0 and Mplus 8.30. Firstly, descriptive statistics and correlation analysis were conducted on the main variables in the study using SPSS 26.0. Secondly, Mplus 8.30 was utilized to establish cross-lagged panel model (CLPM) and random intercept cross-lagged panel model (RI-CLPM), in order to explore the dynamic development relationships between parent-child communication, self-esteem and suicidal ideation at both between-person and within-person levels. The main indicators for evaluating the model fit included χ^2^/*df*, CFI, TLI, RMSEA, and SRMR. When CFI > 0.90, TLI > 0.90, RMSEA < 0.08, and SRMR ≤ 0.08, the model fits well (33).

## 3 Results

### 3.1 Common method bias test

To examine whether common method bias posed a serious concern in this study, Harman’s single-factor test was conducted on the data from each of the four waves. The results indicated that at T1, T2, T3 and T4, the number of factors with eigenvalues greater than 1 was 15, 13, 13, and 12, respectively. the variance explained by the largest single factor was 21.34%, 20.59%, 23.30% and 24.29% at T1, T2, T3 and T4, respectively. These findings suggest that common method bias was not a serious issue in any four waves of data collection.

### 3.2 Descriptive statistics and correlation analysis of variables

Descriptive statistics and correlations are presented in **Table 1**. Father-adolescent communication, mother-adolescent communication, and self-esteem were positively associated with one another across all four waves, whereas each variable was negatively associated with suicidal ideation. All study variables also demonstrated significant temporal stability across waves. Because sex was significantly associated with the study variables, it was included as a control variable in subsequent analyses.

**Table 1:** Descriptive statistics and correlation analysis results of each variable.

Variables	1	2	3	4	5	6	7	8	9	10	11	12	13	14	15	16
1. FAC T1	1															
2. FAC T2	0.63	1														
3. FAC T3	0.66	0.66	1													
4. FAC T4	0.60	0.58	0.71	1												
5. MAC T1	0.73	0.59	0.60	0.54	1											
6. MAC T2	0.61	0.73	0.64	0.57	0.70	1										
7. MAC T3	0.61	0.62	0.75	0.68	0.69	0.73	1									
8. MAC T4	0.54	0.53	0.65	0.77	0.59	0.64	0.66	1								
9. SE T1	0.53	0.45	0.47	0.45	0.59	0.49	0.49	0.42	1							
10. SE T2	0.49	0.57	0.53	0.47	0.57	0.61	0.58	0.48	0.69	1						
11. SE T3	0.48	0.50	0.61	0.49	0.52	0.55	0.64	0.53	0.64	0.72	1					
12. SE T4	0.44	0.46	0.55	0.61	0.44	0.48	0.57	0.61	0.57	0.66	0.72	1				
13. SI T1	–0.56	–0.46	–0.49	–0.45	–0.62	–0.54	–0.53	–0.46	–0.74	–0.64	–0.62	–0.54	1			
14. SI T2	–0.46	–0.56	–0.49	–0.43	–0.53	–0.59	–0.53	–0.44	–0.61	–0.72	–0.62	–0.57	0.69	1		
15. SI T3	–0.46	–0.47	–0.59	–0.47	–0.48	–0.52	–0.62	–0.49	–0.56	–0.64	–0.75	–0.62	0.62	0.67	1	
16. SI T4	–0.42	–0.44	–0.52	–0.58	–0.42	–0.48	–0.53	–0.56	–0.49	–0.57	–0.61	–0.63	0.56	0.61	0.65	1
*M*	64.38	66.23	66.05	65.55	66.13	66.87	67.35	66.76	35.92	36.23	36.52	36.56	28.42	28.48	27.10	28.20
*SD*	15.06	14.79	14.50	14.05	15.18	14.35	14.44	14.13	8.08	8.00	8.18	8.21	10.48	11.68	10.84	11.18

FAC, Father-adolescent communication; MAC, Mother-adolescent communication; SE, Self-esteem; SI, Suicidal ideation. All correlation coefficients are significant at 0.001 (two-tailed).

### 3.3 Longitudinal measurement invariance test

To ensure that the measurement instruments consistently and validly assessed the target constructs across different time points, longitudinal measurement invariance tests were conducted. Given that the chi-square test is highly sensitive to sample size, changes in fit indices between nested models were employed to evaluate measurement equivalence. Criteria for acceptable model invariance were defined as ΔCFI ≤ 0.01, ΔSRMR ≤ 0.03, and ΔRMSEA ≤ 0.015 (34). As shown in **Table 2**, parent-child communication, self-esteem, and suicidal ideation measured in this study all met the criteria for cross-time measurement invariance, indicating that the instruments possess satisfactory measurement invariance (35).

**Table 2:** Longitudinal measurement invariance tests for each variable.

Variables	Model	χ^2^	*df*	CFI	TLI	SRMR	RMSEA	ΔCFI	ΔSRMR	ΔRMSEA
Father-adolescent communication	Configural invariance	9212.51	2969	0.836	0.826	0.086	0.045			
	Metric invariance	9673.19	3018	0.826	0.817	0.091	0.046			
	Scalar invariance	10352.29	3087	0.810	0.805	0.091	0.048			
Mother-adolescent communication	Configural invariance	9909.87	2969	0.822	0.810	0.093	0.046			
	Metric invariance	10243.29	3018	0.815	0.806	0.100	0.046	–0.007	–0.004	0.000
	Scalar invariance	10965.96	3087	0.811	0.800	0.075	0.048	–0.004	–0.006	0.002
Self-esteem	Configural invariance	2597.43	711	0.920	0.912	0.077	0.048			
	Metric invariance	2708.78	738	0.916	0.911	0.080	0.049	–0.004	0.003	0.001
	Scalar invariance	2840.96	765	0.912	0.910	0.080	0.049	–0.004	0.000	0.000
Suicidal ideation	Configural invariance	4030.31	1400	0.928	0.921	0.081	0.041			
	Metric invariance	4180.68	1410	0.924	0.917	0.082	0.042	–0.004	0.001	0.001
	Scalar invariance	4565.12	1421	0.914	0.907	0.085	0.044	–0.010	0.003	0.002

### 3.4 The CLPM and RI-CLPM of father-adolescent communication, self-esteem and suicidal ideation

Sex was included as a control variable in both models. The CLPM showed mixed model fit indices (χ^2^ = 401.65, *df* = 27, RMSEA = 0.116, CFI = 0.957, TLI = 0.877, SRMR = 0.062), with some indices indicating acceptable fit but RMSEA suggesting suboptimal fit.

**Figure 1:**
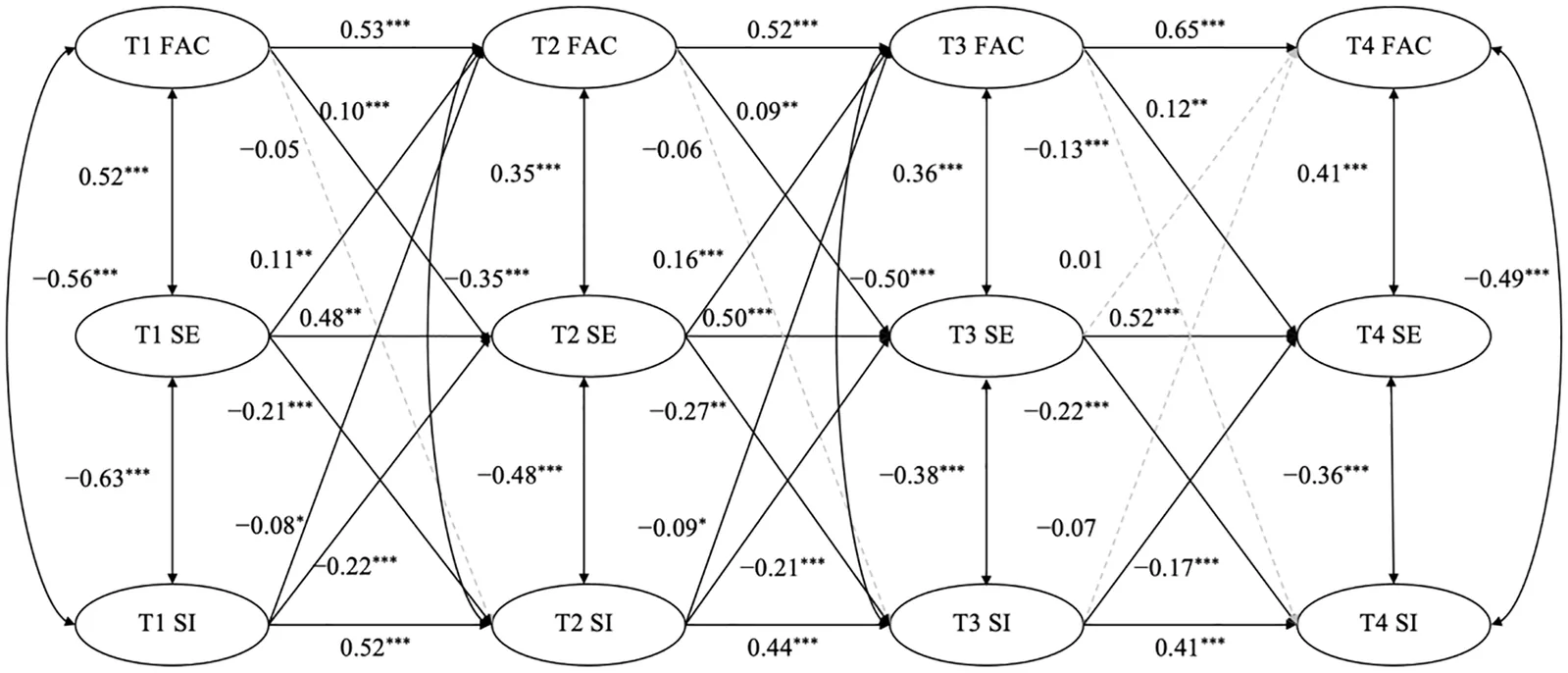
CLPM of father-adolescent communication, self-esteem and suicidal ideation. CLPM, Cross-lagged panel model; FAC, Father-adolescent communication; SE, Self-esteem; SI, Suicidal ideation; T1, Time 1; T2, Time 2; T3, Time 3. Dotted lines indicate nonsignificant paths. All coefficients shown in the figure are standardized path coefficients. ^*^p < 0.05, ^**^p < 0.01, ^***^p < 0.001.

As shown in **Figure 1**, significant autoregressive effects were observed for all variables, indicating temporal stability across waves. Father-adolescent communication positively predicted subsequent self-esteem across waves (all *ps* < 0.01), whereas self-esteem positively predicted later father-adolescent communication during earlier waves (all *ps* < 0.01). Suicidal ideation negatively predicted subsequent father-adolescent communication during earlier waves (*ps* < 0.05). In addition, self-esteem and suicidal ideation showed reciprocal longitudinal associations. Mediation analyses further indicated significant indirect effects of self-esteem. Specifically, father-adolescent communication at T1 indirectly predicted lower suicidal ideation at T3 through self-esteem at T2 (indirect effect = −0.020, *p* = 0.003), and father-adolescent communication at T2 indirectly predicted lower suicidal ideation at T4 through self-esteem at T3 (indirect effect = −0.015, *p* = 0.020), suggesting that self-esteem partially mediated the longitudinal association between father-adolescent communication and suicidal ideation.

Given that limitations of CLPM in separating between-person and within-person effects, the RI-CLPM was further conducted, which showed good model fit (χ² = 37.42, *df* = 21, RMSEA = 0.027, CFI = 0.998, TLI = 0.993, SRMR = 0.025) and fit the data better than the traditional CLPM.

**Figure 2:**
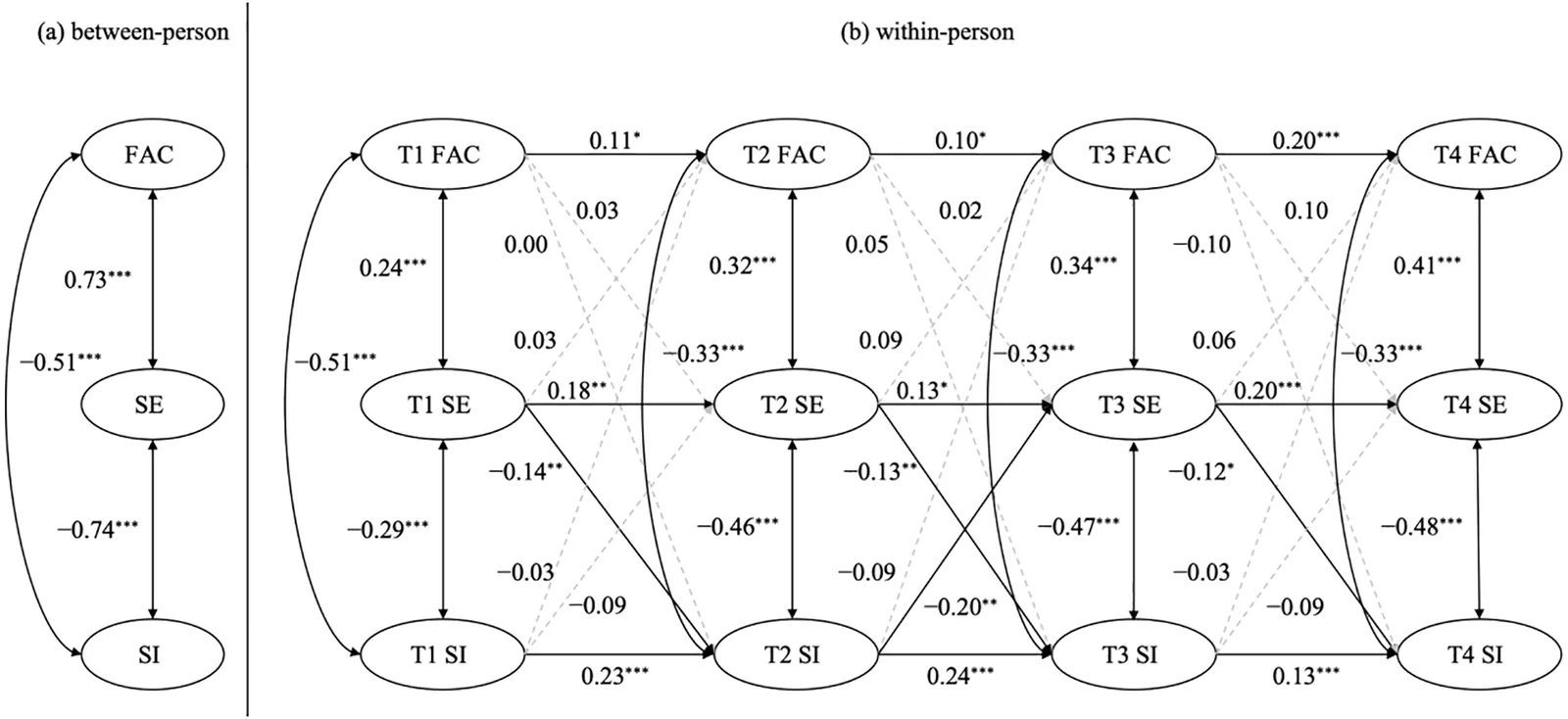
RI-CLPM of father-adolescent communication, self-esteem and suicidal ideation. RI-CLPM, Random-intercept cross-lagged panel model; FAC, Father-adolescent communication; SE, Self-esteem; SI, Suicidal ideation; T1, Time 1; T2, Time 2; T3, Time 3. Dotted lines indicate nonsignificant paths. All coefficients shown in the figure are standardized path coefficients. ^*^p < 0.05, ^**^p < 0.01, ^***^p < 0.001. **(a)** represents the between-person level, and **(b)** represents the within-person level.

As shown in **Figure 2**, the RI-CLPM examined within-person associations by separating stable between-person differences from within-person fluctuations over time. At the within-person level, significant autoregressive effects remained, indicating that deviations from an individual’s typical level tended to persist across waves. Self-esteem consistently predicted subsequent decreases in suicidal ideation (all *ps* < 0.05), suggesting that adolescents reporting higher-than-usual self-esteem were less likely to experience increases in suicidal ideation over time. Evidence for the reverse association was more limited, with suicidal ideation significantly predicting lower subsequent self-esteem only from T2 to T3 (*p* = 0.001). Although the within-person path from self-esteem to suicidal ideation was significant, the indirect effects were not supported because the product of the within-person paths from father-adolescent communication to self-esteem and from self-esteem to suicidal ideation did not significantly differ from zero, with all bootstrap 95% confidence intervals included zero.

### 3.5 The CLPM and RI-CLPM of mother-adolescent communication, self-esteem and suicidal ideation

Sex was included as a control variable in both models. The fit indices for the mother-adolescent communication models were similar to those reported for the father-adolescent models: the CLPM yielded: χ² = 361.05, *df* = 27, RMSEA = 0.109, CFI = 0.964, TLI = 0.897, SRMR = 0.047, with RMSEA again exceeding the conventional threshold of 0.10, indicating suboptimal fit.

**Figure 3:**
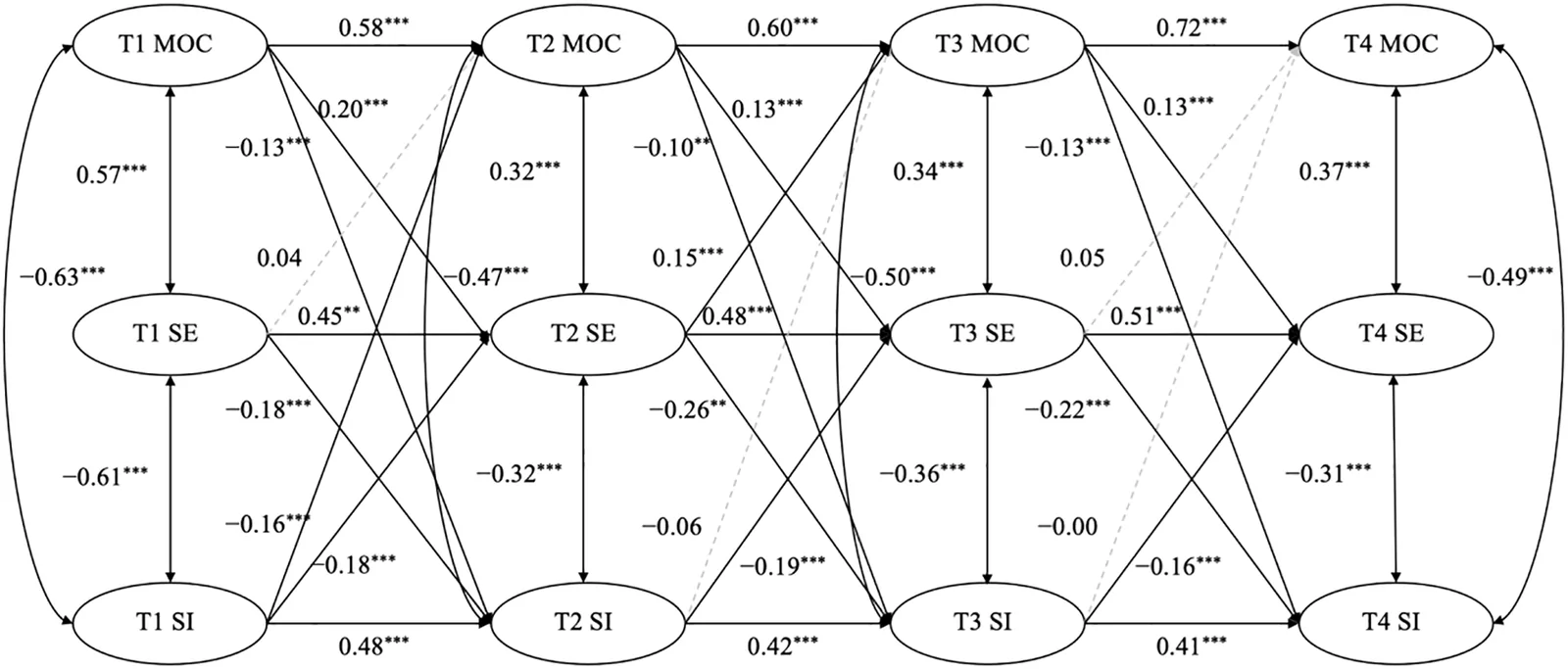
CLPM of mother-adolescent communication, self-esteem and suicidal ideation. CLPM, Cross-lagged panel model; MOC, Mother-adolescent communication; SE, Self-esteem; SI, Suicidal ideation; T1, Time 1; T2, Time 2; T3, Time 3. Dotted lines indicate nonsignificant paths. All coefficients shown in the figure are standardized path coefficients. ^*^p < 0.05, ^**^p < 0.01, ^***^p < 0.001.

As shown in **Figure 3**, significant autoregressive effects were observed for all variables, indicating temporal stability across waves. Mother-adolescent communication positively predicted subsequent self-esteem and negatively predicted later suicidal ideation across all waves (all *ps* < 0.01). Self-esteem positively predicted subsequent mother-adolescent communication during earlier waves (*p* < 0.001), whereas suicidal ideation negatively predicted later mother-adolescent communication (*ps* < 0.05). In addition, self-esteem and suicidal ideation showed reciprocal longitudinal associations. Mediation analyses further revealed significant indirect effects of self-esteem. Specifically, the indirect effect from T1 mother-adolescent communication to T3 suicidal ideation through T2 self-esteem was significant (indirect effect = −0.037, *p* < 0.001). Similarly, the indirect effect from T2 mother-adolescent communication to T4 suicidal ideation through T3 self-esteem was also significant (indirect effect = −0.021, *p* = 0.004).

In contrast, the RI-CLPM showed substantially better fit (χ² = 37.42, *df* = 21, RMSEA = 0.031, CFI = 0.998, TLI = 0.991, SRMR = 0.022), confirming its superiority over the CLPM.

**Figure 4:**
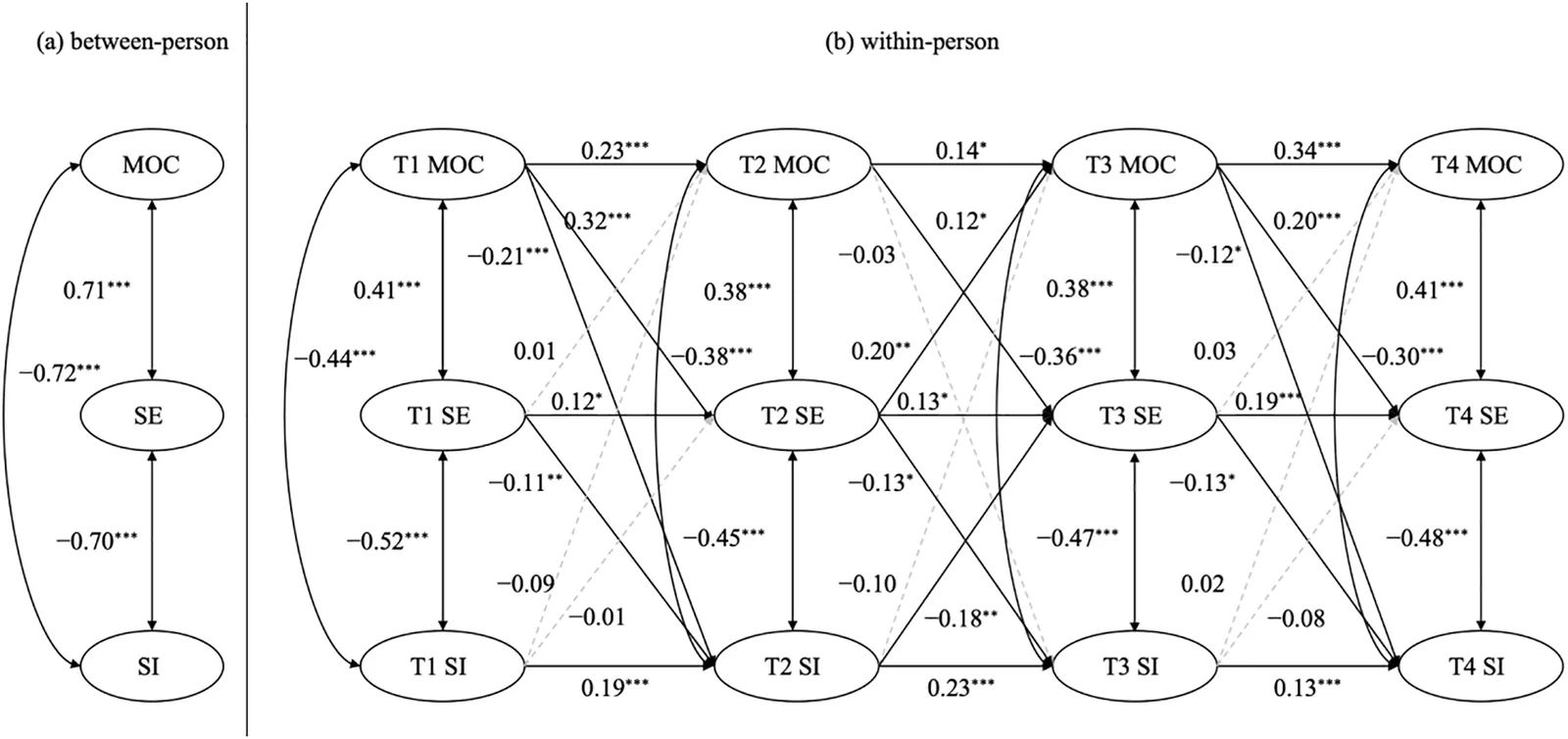
RI-CLPM of mother-adolescent communication, self-esteem and suicidal ideation. RI-CLPM, Random-intercept cross-lagged panel model; MOC, Mother-adolescent communication; SE, Self-esteem; SI, Suicidal ideation; T1, Time 1; T2, Time 2; T3, Time 3. Dotted lines indicate nonsignificant paths. All coefficients shown in the figure are standardized path coefficients. ^*^p < 0.05, ^**^p < 0.01, ^***^p < 0.001. **(a)** represents the between-person level, and **(b)** represents the within-person level.

As shown in **Figure 4**, the RI-CLPM examined within-person associations by separating stable between-person differences from within-person fluctuations over time. At the within-person level, significant autoregressive effects remained, indicating that deviations from adolescents’ typical levels of mother-adolescent communication, self-esteem, and suicidal ideation tended to persist across waves. Mother-adolescent communication consistently predicted subsequent increases in self-esteem (all *ps* < 0.05), suggesting that adolescents reporting higher-than-usual communication with their mothers also tended to report higher-than-usual self-esteem over time. Mother-adolescent communication additionally predicted subsequent decreases in suicidal ideation at selected time intervals (*ps* < 0.05), indicating that increases in mother-adolescent communication may provide short-term protection against suicidal ideation. Self-esteem consistently predicted subsequent decreases in suicidal ideation (all *ps* < 0.05), whereas evidence for reverse associations was more limited. Specifically, self-esteem positively predicted subsequent mother-adolescent communication only from T2 to T3 (*p* = 0.002). Bootstrap analyses further showed that none of the indirect effects of self-esteem between mother-adolescent communication and suicidal ideation reached statistical significance. Bootstrap analyses further showed that none of the within-person indirect effects of self-esteem between mother-adolescent communication and suicidal ideation reached statistical significance. Although mother-adolescent communication significantly predicted subsequent changes in self-esteem and self-esteem significantly predicted later suicidal ideation, the combined indirect effects were not statistically different from zero, as indicated by bootstrap 95% confidence intervals that included zero.

## 4 Discussion

This study used four-wave longitudinal data and applied both cross-lagged panel models (CLPM) and random-intercept cross-lagged panel models (RI-CLPM). By separating stable between-person differences from within-person changes over time, these models provide a clearer understanding of the longitudinal relationships among parent-adolescent communication, self-esteem, and suicidal ideation.

### 4.1 The relationship between parent-adolescent communication and suicidal ideation

At the between-person level, both father-adolescent and mother-adolescent communication were negatively associated with adolescent suicidal ideation, indicating that adolescents who generally reported better communication with parents tended to report lower levels of suicidal ideation. This finding is consistent with family systems theory, which emphasizes the importance of family interactions for adolescent development. Notably, mother-adolescent communication showed a stronger association with suicidal ideation than father-adolescent communication, consistent with previous findings (36, 37). This difference may reflect mothers’ continuing role as a primary source of emotional support in adolescents’ daily lives (38).

At the within-person level, however, only fluctuations in mother-adolescent communication predicted subsequent decreases in suicidal ideation. Specifically, adolescents reporting higher-than-usual communication with their mothers subsequently experienced lower suicidal ideation, whereas within-person effects of father-adolescent communication were not significant. These findings suggest that mother-adolescent communication may provide more immediate protection against suicidal ideation. Mothers are often more involved in emotional expression and daily caregiving, and increased emotional responsiveness may therefore exert more direct short-term effects on adolescents’ emotional well-being (39).

The non-significant within-person effect of father-adolescent communication should be interpreted with caution. This finding does not suggest that father-adolescent communication is unimportant; rather, it indicates that temporary changes in paternal communication were not associated with subsequent changes in suicidal ideation. This pattern differs from the between-person findings, where adolescents with generally higher levels of father-adolescent communication reported lower suicidal ideation, suggesting that paternal communication may contribute to adolescent adjustment through relative stable developmental processes. Several explanations may account for this finding. First, previous research has suggested that fathers may contribute to adolescents’ development of autonomy, competence, and self-regulation (40, 41). These psychological resources are likely to be shaped by sustained patterns of interaction rather than short-term fluctuations in communication. Thus, paternal influences may unfold over longer development periods rather than immediately following temporary changes in communication quality (42, 43).

Besides, cultural and family-role factors may also contribute to this pattern. Although fathers have increasingly taken on more family responsibilities with sociocultural changes in recent years, mother continue to provide more daily care for children, partly due to the influence of traditional gender roles (44). As a result, maternal communication may be more closely embedded in adolescents’ daily emotional exchanges, making short-term changes in maternal communication more likely to influence immediate psychological states. In contrast, temporary improvements or deteriorations in paternal communication may represent smaller deviations from adolescents’ typical experiences and may therefore have limited within-person effects.

Future studies incorporating more frequent assessments and distinguishing different dimensions of paternal communication (e.g., emotional support, autonomy support, and instrumental guidance) are needed to further clarify the conditions under which paternal communication may influence adolescent suicidal ideation.

### 4.2 The relationship between self-esteem and suicidal ideation

The CLPM revealed reciprocal associations between self-esteem and suicidal ideation at the between-person level. Adolescents characterized by chronically lower self-esteem tended to report higher suicidal ideation, whereas greater suicidal ideation predicted subsequent reductions in self-esteem. These findings support both the vulnerability model and the scar model (45).

After separating stable between-person differences, the RI-CLPM revealed a more nuanced pattern. Self-esteem consistently predicted subsequent suicidal ideation at the within-person level, whereas reverse effects from suicidal ideation to self-esteem were comparatively limited (46). This finding suggests that decreases in self-esteem reliably increase vulnerability to suicidal ideation regardless of whether self-esteem is conceptualized as a stable trait or short-term fluctuation. In contrast, isolated episodes of suicidal ideation may be insufficient to substantially alter adolescents’ self-evaluations.

This pattern may also reflect developmental characteristics of adolescence. Self-esteem gradually shifts from relative instability toward greater consolidation during this period (47). Consequently, temporary psychological distress may not immediately alter core self-evaluations, whereas sustained negative experiences may be required before scar effects emerge. Previous longitudinal evidence similarly suggests that scar effects become more pronounced during later developmental stages (48).

### 4.3 The longitudinal mediating role of self-esteem

The findings revealed different patterns of mediation across analytical levels. At the between-person level, self-esteem significantly mediated the associations between both father- and mother-adolescent communication and suicidal ideation, with complete mediation observed for father-adolescent communication. These findings suggest that parent-adolescent communication may influence suicidal ideation not only directly but also indirectly through shaping adolescents’ self-evaluations. Consistent with previous research, supportive parent-adolescent interactions characterized by acceptance, respect, and emotional support may contribute to the development of more positive self-perceptions and psychological resources, which in turn reduce vulnerability to suicidal ideation (49–51). The complete mediation observed for father-adolescent communication further suggests that paternal communication may be particularly relevant to suicidal ideation through its association with adolescents’ self-esteem, although other pathways may also contribute to this relationship.

At the within-person level, however, none of the mediating effects of self-esteem reached statistical significance. The discrepancy between CLPM and RI-CLPM findings can be attributed to the different variance components captured by the two models. The CLPM does not separate between-person differences from within-person fluctuations, and therefore significant indirect effects may partly reflect that adolescents with generally better parent-adolescent communication also tend to have higher self-esteem and lower suicidal ideation. By contrast, the RI-CLPM isolates within-person processes by controlling for time-invariant individual differences, and therefore tests whether temporary deviations in communication predict subsequent changes in self-esteem and suicidal ideation. The non-significant indirect effects in the RI-CLPM suggests that short-term fluctuations in parent-adolescent communication may not consistently translate into changes in self-esteem that are large enough to alter suicidal ideation.

Several substantive explanations may further account for the absence of within-person mediation. First, temporary improvements in self-esteem, even if they occur, may be offset by concurrent proximal stressors, such as academic pressure or peer difficulties, that are common during adolescence (52, 53). Second, communication, self-esteem, and suicidal ideation may operate on different timescales: changes in self-esteem may require longer periods to consolidate before they can exert detectable influences on suicidal ideation, whereas the protective effects of mother–adolescent communication on suicidal ideation may be mediated by more immediate mechanisms (e.g., emotional regulation or perceived support) rather than through self-esteem.

Taken together, the findings support a dual-pathway explanation. Parent-adolescent communication appears to influence suicidal ideation through long-term pathways involving self-esteem while also exerting more immediate effects, particularly through mother-adolescent communication. These findings have some practical implications for adolescent suicide prevention and family-based interventions. First, parent-adolescent communication may represent an important and modifiable target for reducing suicidal ideation. Given the significant within-person association between mother-adolescent communication and subsequent changes in suicidal ideation, prevention programs may benefit from helping mothers develop more supportive communication strategies, including active listening, emotional validation, and open discussion of adolescents’ difficulties. Second, the findings regarding self-esteem provide implications for prevention efforts (54). Although self-esteem mediated the association between parent-adolescent communication and suicidal ideation in the CLPM, this effects was not supported at the within-person level in RI-CLPM. This suggests that self-esteem may function primarily as a relatively stable psychological resource underlying long-term differences in suicidal ideation rather than as an immediate mechanism linking short-term changes in communication to suicidal ideation. Thus, self-esteem promotion may be valuable as part of long-term prevention strategies. Third, the differential patterns of father- and mother-adolescent communication suggest that family interventions should consider the distinct roles of parents. Although father-adolescent communication did not demonstrate significant within-person associations with suicidal ideation, it was associated with adolescents’ self-esteem and suicidal ideation at the between-person level in CLPM. Therefore, fathers should also be encouraged to participate in prevention programs by providing consistent support, encouragement, and autonomy-related guidance.

### 4.4 Limitations and future directions

Several limitations should be acknowledged. First, participants were recruited from two middle schools in Hunan Province, China, which may limit the generalizability of the findings to adolescents from other regions, cultural backgrounds, or educational contexts. Cultural characteristics of Chinese adolescents, including family values and parent-child interaction patterns, may influence the associations among parent-adolescent communication, self-esteem, and suicidal ideation. Future studies should replicate these findings using more geographically, culturally, and socially samples. Second, all variables were assessed using adolescent self-report measures. Although self-reports are commonly used to assess adolescents’ subjective experiences, they may be influenced by recall bias, social desirability, and individual perceptions. In addition, adolescent reports may not fully capture the dynamic and reciprocal nature of parent-adolescent communication. Future research should adopt multi-informant designs (e.g., parent reports) and integrate multiple assessment methods, such as behavioral observations or ecological momentary assessment, to obtain a more comprehensive understanding of these relationships. Third, although the RI-CLPM controls for time-invariant individual differences, the present study did not include some important time-varying contextual factors, such as peer relationships and academic stress (55, 56), which may influence adolescents’ suicidal ideation over time. Future studies should incorporate these factors to further clarify the complex developmental mechanisms underlying adolescent suicidal ideation.

## 5 Conclusion

This study examined the longitudinal associations among parent-adolescent communication, self-esteem, and suicidal ideation using both CLPM and RI-CLPM. The findings suggest that mother-adolescent communication showed both short-term and long-term protective effects on suicidal ideation, whereas father-adolescent communication mainly showed indirect long-term effects through self-esteem. These findings highlight the importance of distinguishing between fathers’ and mothers’ roles in adolescent mental health.

## Data Availability

The raw data supporting the conclusions of this article will be made available by the authors, without undue reservation.
